# A Rare Case of Grynfeltt-Lesshaft Hernia in an Elderly Patient

**DOI:** 10.7759/cureus.82517

**Published:** 2025-04-18

**Authors:** Gowtham Ganesan, Alexander Mecheri Antony

**Affiliations:** 1 General Surgery, Sree Balaji Medical College and Hospital, Chennai, IND; 2 Surgery, Sree Balaji Medical College and Hospital, Chennai, IND

**Keywords:** grynfelt-lesshaft hernia, grynfelt-lesshaft hernia repair, hernia, lumbar hernia, open hernia surgery

## Abstract

Lumbar hernia, a rare and challenging condition in which abdominal contents protrude through defects in the posterolateral abdominal wall, has been recognized since the 17th century. It typically involves two key anatomical regions: the superior lumbar triangle (Grynfeltt-Lesshaft) and the inferior lumbar triangle (Petit). Due to the limited number of documented cases, a deep understanding of the specific anatomy and weaknesses in the lumbar region is crucial for accurate diagnosis and management.

Diagnosing lumbar hernias can be difficult, often leading to misdiagnosis as lipomas or abscesses. Clinically, hernias present as bulges that tend to become more prominent with physical activity. A contrast-enhanced CT scan is essential for preoperative evaluation, though certain anatomical variations can complicate diagnosis. Surgical options include traditional open repairs and minimally invasive laparoscopic techniques, with mesh placement being commonly used. The choice of approach must be individualized, considering the hernia’s size, contents, and the patient’s overall condition. Despite advancements, achieving optimal outcomes remains challenging due to the complexity of the involved anatomy.

Whether congenital or acquired, a Grynfeltt-Lesshaft hernia requires heightened awareness and a solid understanding of lumbar anatomy for early diagnosis. In resource-limited settings, open repair may be the most practical surgical approach. Effective management relies on a comprehensive clinical evaluation and the selection of the appropriate surgical technique to minimize recurrence and ensure the best possible patient outcomes.

## Introduction

Lumbar hernias are relatively rare compared to other types of hernias. According to Hafner et al. [1], a general surgeon may encounter a lumbar hernia only once in their entire career. However, with the increasing incidence of traumatic causes and advancements in diagnostic imaging, lumbar hernias are being diagnosed more frequently in modern clinical practice.

The concept of a lumbar hernia was first introduced by P. Barbette in 1672, and the first documented case was recorded by R.J.C. Garangeot in 1731 [2]. Since then, approximately 300 cases have been documented [3].

The lumbar region is defined by the 12th rib superiorly, the iliac crest inferiorly, the erector spinae muscles medially, and the external oblique muscle (EOM) laterally. Two potential weak spots, the superior and inferior lumbar gaps, are where lumbar hernias can occur [4]. The inferior lumbar area is surrounded by the EOM on the side, the latissimus dorsi muscle (LDM) medially, and the iliac crest below. The superior lumbar area is shaped like an inverted triangle. The EOM and LDM form the roof of the superior lumbar triangle, while the internal oblique muscle forms the anterior boundary, and the 12th rib and lower edge of the serratus posterior inferior muscle form its base [5]. While it was previously thought that inferior lumbar hernias were more common, recent studies show that the superior lumbar triangle is actually where hernias most frequently occur [6].

Lumbar hernias can also be categorized based on their origin. They may be acquired or congenital. There are two types of acquired hernias: primary (spontaneous) and secondary. Secondary lumbar hernias can result from trauma, infections, or previous surgical procedures [7]. Of these, primary spontaneous hernias are the least common [8].

## Case presentation

**Figure 1:**
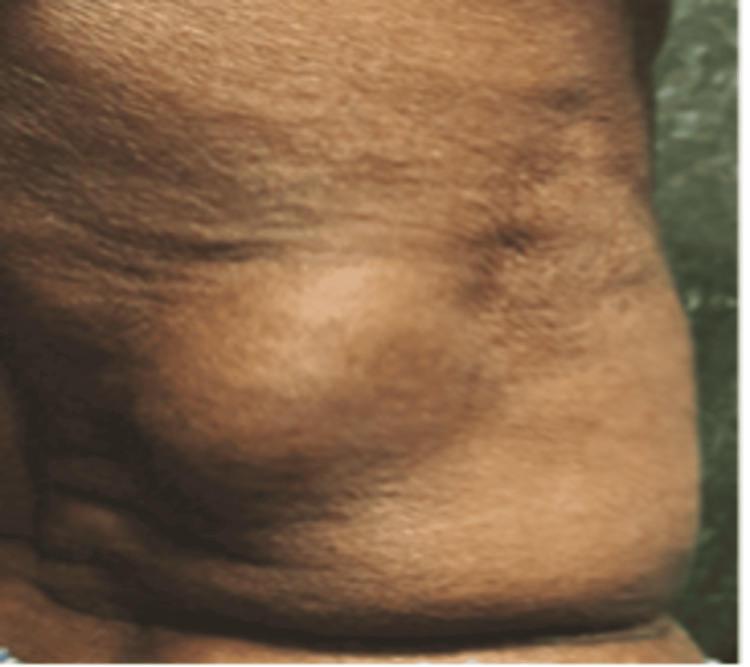
A pre-operative image displaying swelling in the right lumbar region.

A 65-year-old male presented with swelling in his right lumbar region. He had no history of medical comorbidities or excessive physical strain. The BMI of the patient is 23.7 kg/m^2^. On physical examination, a 10 × 8 cm swelling was observed beneath the twelfth rib in the right lumbar area. The swelling was ovoid, soft, painless, and exhibited an impulsive expansion when coughing. It was non-pulsatile and became less noticeable when the patient was lying down, but became visible upon standing and coughing (Figure 1).

**Figure 2:**
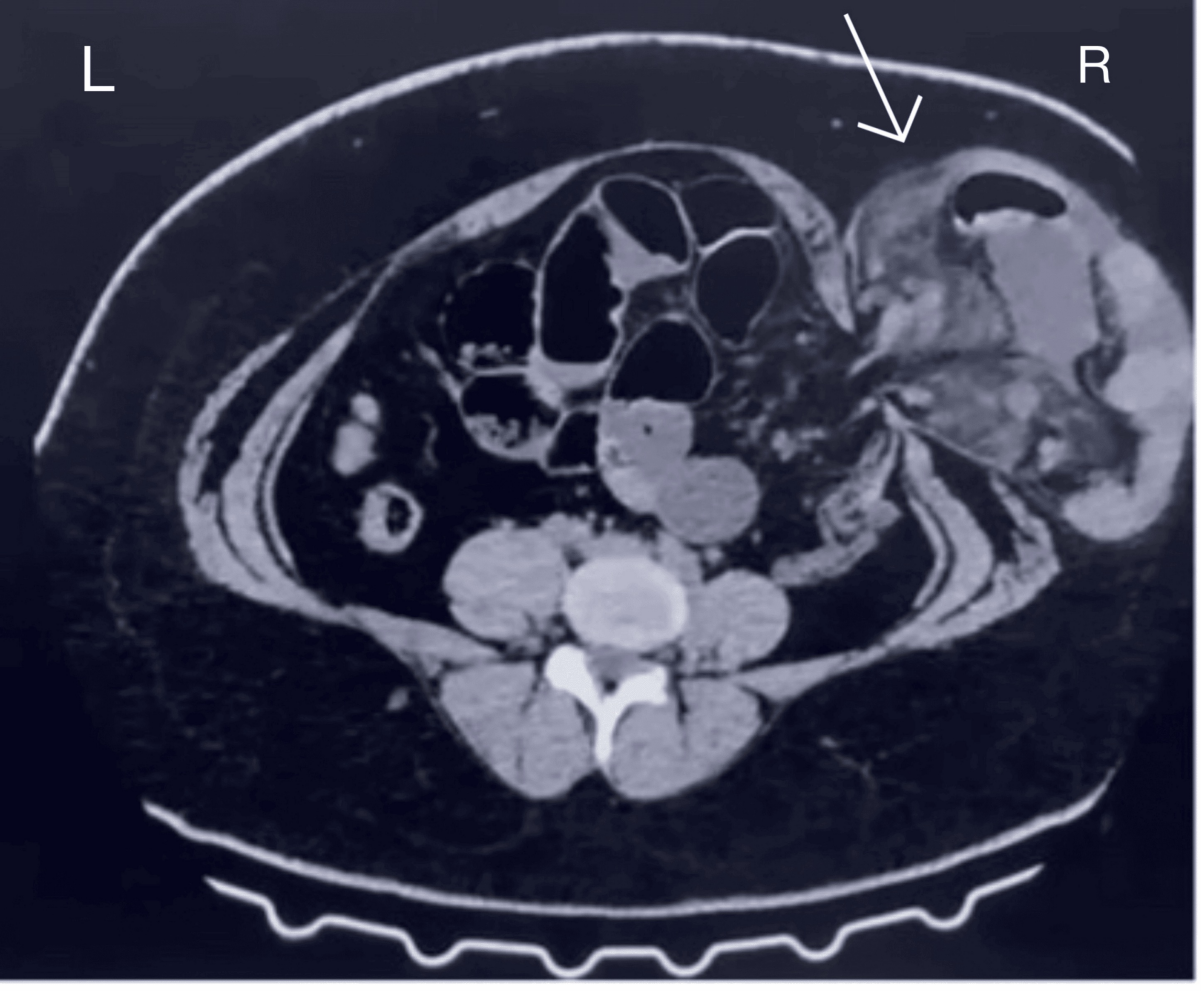
Contrast-enhanced CT abdomen showing herniation of bowel loops with omentum in the right lumbar region (white arrow).

A distinct coughing impulse was present. A contrast-enhanced CT scan (CECT) revealed that bowel loops and omentum had emerged from a defect in the right posterolateral body wall (Figure 2).

The patient was positioned in a left lateral decubitus position under general anesthesia to optimize access to the right lumbar region. A transverse incision approximately 5 cm above the right anterior superior iliac spine (ASIS) was marked and made, corresponding to the site of the herniation. The incision extended laterally over the edematous swelling and was deepened carefully through the subcutaneous tissue using electrocautery, taking care to preserve surrounding structures.

**Figure 3:**
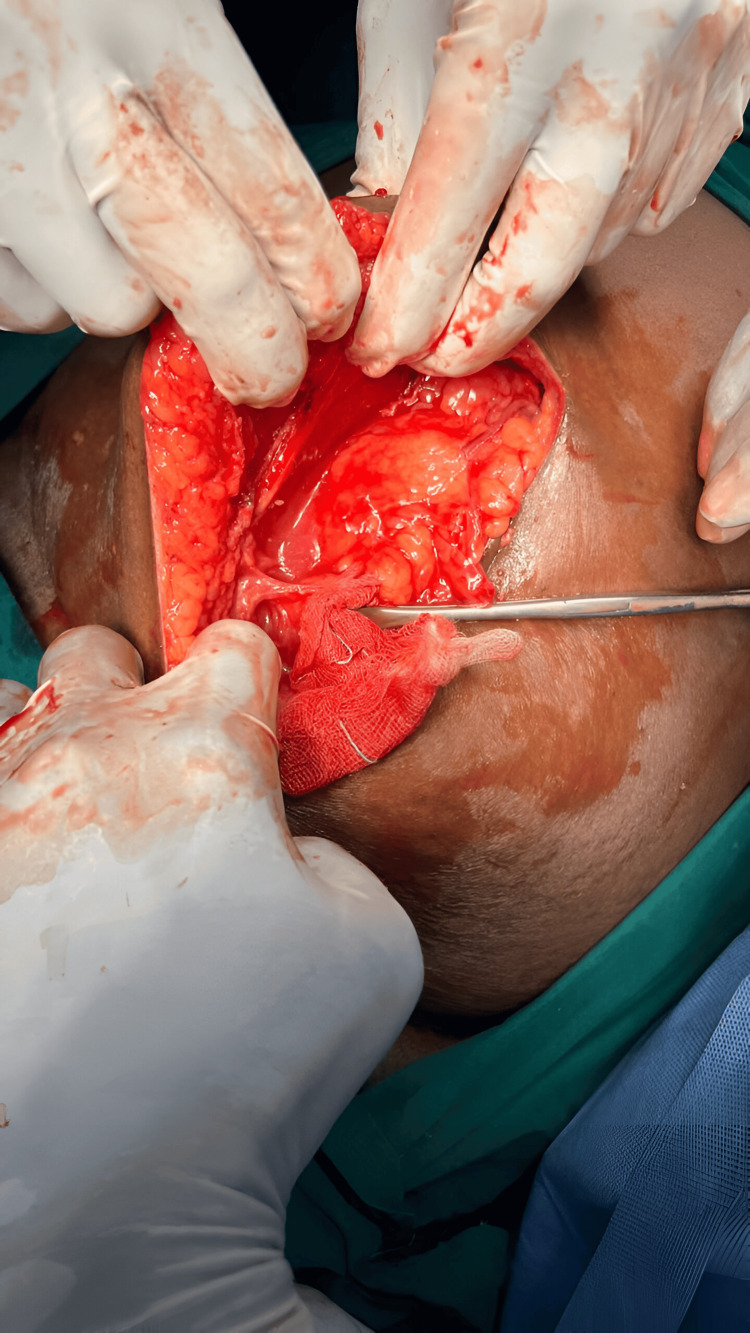
An intraoperative image showing the defect.

Upon reaching the muscular layer, the external oblique, internal oblique, and transversus abdominis muscles were sequentially dissected and retracted to expose the hernial sac and the contents protruding through the defect (Figure 3).

The hernial sac was carefully opened, and contents, including bowel loops and omentum, were inspected for viability. Since there were no signs of strangulation or ischemia, the contents were gently reduced back into the peritoneal cavity.

**Figure 4:**
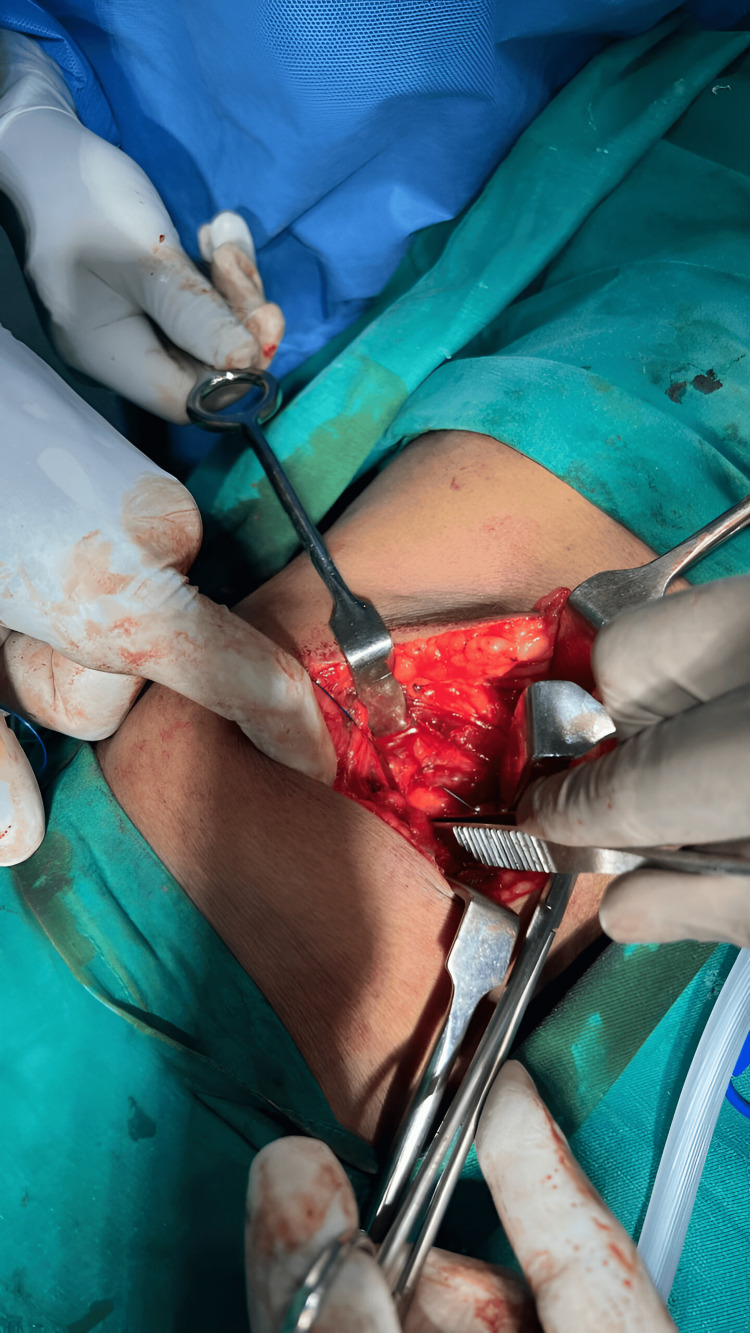
The defect is being closed primarily with Prolene sutures using a continuous technique.

The hernia defect, located within the superior lumbar triangle, was measured and found to be of significant size. It was closed primarily using a continuous non-absorbable Prolene (Ethicon Inc., Raritan, NJ) suture to bring the edges of the muscular and fascial layers together, thus reconstituting the integrity of the posterior abdominal wall (Figure 4).

**Figure 5:**
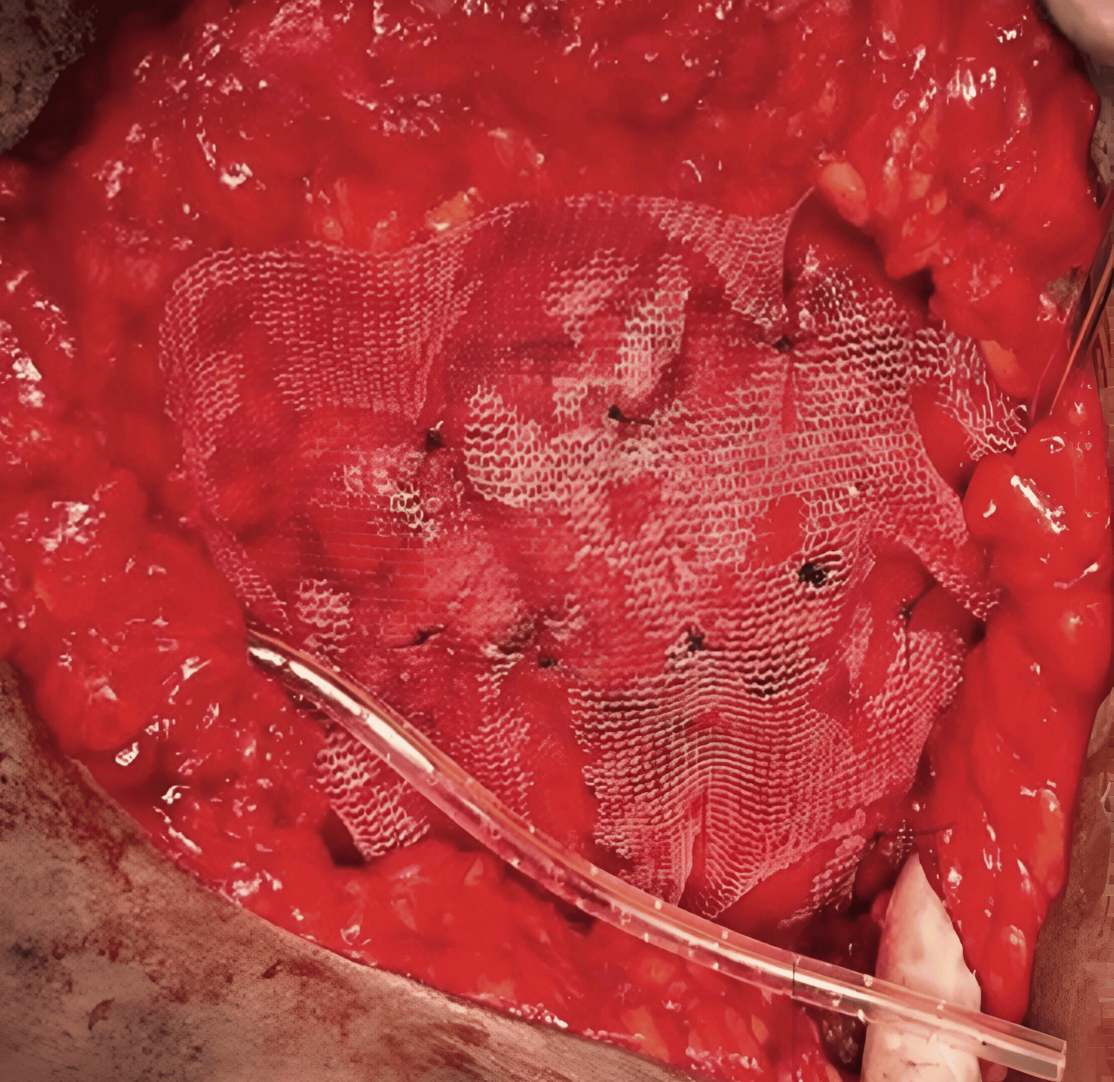
Intraoperative image of Prolene mesh being placed over the defect.

To reinforce the repair and reduce the risk of recurrence, a sterile Prolene mesh of size 15 × 15 cm was placed in an onlay fashion over the closed defect. The mesh was secured with multiple interrupted Prolene sutures anchored to the strong surrounding musculature, including the transversus abdominis, internal oblique, and the periosteum of the 12th rib and iliac crest, ensuring a tension-free repair (Figure 5).

**Figure 6:**
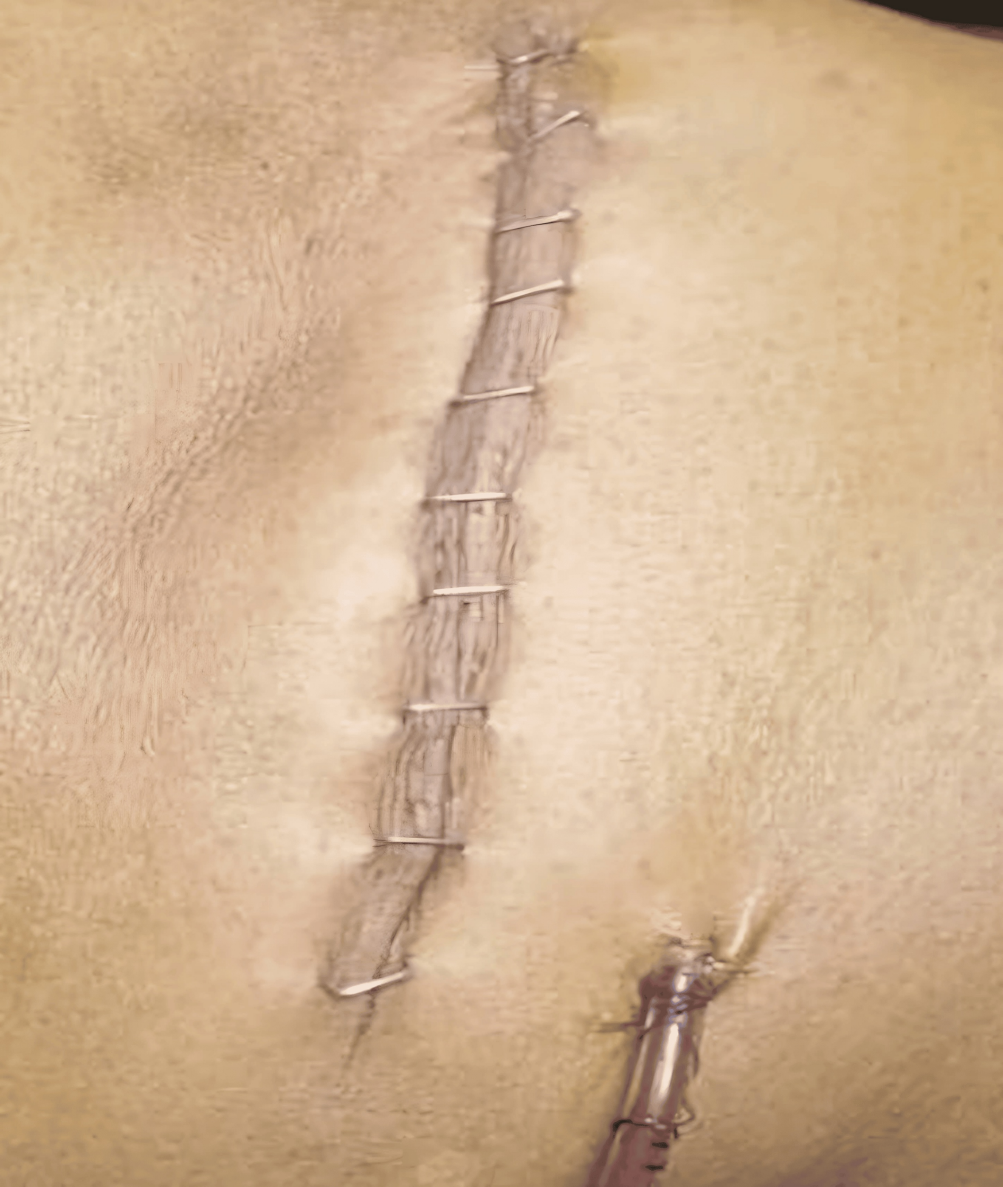
Image showing skin closure using skin staplers.

A closed-suction drain of size 14FG (Romovac, Romsons Group Private Limited, Agra, India) was placed in the surgical field to prevent seroma or hematoma formation and fixed securely to the skin. Hemostasis was confirmed, and the wound was closed in layers. The muscle and fascial layers were approximated with absorbable sutures, followed by subcutaneous closure. Skin closure was achieved using surgical skin staplers (Figure 6), and a sterile dressing was applied.

The postoperative period was uneventful. The drain was removed on the third postoperative day, and the patient was mobilized early with the help of physiotherapy. Follow-up at one year revealed no recurrence or complications, and the patient reported satisfactory functional recovery.

## Discussion

Primary lumbar hernias typically present as a palpable swelling that expands during coughing. When the patient lies down, the swelling may disappear, and the hernia is often reducible. Patients commonly report vague back or abdominal discomfort, which can sometimes be mistaken for sciatica. In some cases, lumbar hernias may lead to complications like urinary or intestinal obstruction, potentially causing hydronephrosis. Differential diagnoses to consider include lipomas, fibromas, abscesses, hematomas, intra-abdominal or retroperitoneal tumors, and panniculitis [9].

X-rays are generally not useful for diagnosing lumbar hernias, with CT scans being the preferred diagnostic tool. In certain situations, MRI may also be helpful for diagnosis and surgical planning [10].

Open (conventional) repair has a recurrence rate of around 10-20%, higher if mesh is not used. Laparoscopic repair has a recurrence rate of typically less than 5% due to better visualization and tension-free mesh placement (Table 1).

**Table 1:** The recurrence rates for lumbar hernia repairs vary based on the surgical technique, surgeon expertise, and patient-specific factors

Technique	Recurrence rate
Open (conventional)	10-20%
Laparoscopic	0-5%

Approximately 91% of lumbar hernias are non-emergent, with only 9% requiring emergency surgery. Surgery remains the only effective treatment and should be performed promptly to prevent complications. Historically, muscle flaps from the latissimus dorsi, gluteus maximus and medius, and fascia lata were used for lumbar hernia repair. However, these methods had high recurrence rates due to strain on the repair and the weakness of the fascia. The defect was eventually closed using synthetic meshes, such as Marlex (Chevron Phillips Chemical, The Woodlands, TX) or polypropylene [11].

In the Dowd-Ponka approach, the hernia is exposed, its contents are reduced into the abdomen, and the defect is covered with prosthetic mesh sutured to the lumbar periosteum, latissimus dorsi, and external oblique. A gluteal fascial flap is then applied to approximate the latissimus dorsi and EOMs over the lesion [12]. The first transabdominal laparoscopic technique was demonstrated by Burick and Parascandola in 1996 [5], while Woodward et al. introduced the complete extraperitoneal technique using a balloon dissector in 1999 [4]. A 2005 study comparing conventional and laparoscopic techniques for lumbar hernia repair found that the laparoscopic method resulted in significantly lower morbidity, shorter hospital stays, reduced analgesic use, and faster recovery [7].

## Conclusions

A rare condition known as a Grynfeltt-Lesshaft hernia can be either congenital or acquired. Symptoms typically include lower back pain and a lump that diminishes when the patient lies down, which suggests a lumbar hernia. In low-resource settings, where many patients cannot afford a CT scan (the gold standard for diagnosis), surgeons must enhance their ability to diagnose this condition. Among the various surgical techniques available, the open approach is the most cost-effective in these settings. Knowledge of the lumbar triangle's anatomy is crucial for prompt diagnosis. To confirm the diagnosis, a contrast-enhanced CT (CECT) scan is essential. For this rare condition, open mesh repair provides a simple, safe, and efficient alternative to laparoscopic surgery.
